# Suppression of VEGF-induced angiogenesis and tumor growth by *Eugenia jambolana, Musa paradisiaca,* and *Coccinia indica* extracts

**DOI:** 10.1080/13880209.2017.1307422

**Published:** 2017-04-03

**Authors:** Harsha Raj M., Debidas Ghosh, Rita Banerjee, Bharathi P. Salimath

**Affiliations:** a Department of Studies in Biotechnology, Molecular Oncology Lab, University of Mysore, Mysore, India;; b Department of Bio-Medical Laboratory Science & Management, Vidyasagar University, Midnapore, West Bengal, India;; c Department of Science & Technology, Government of India, New Delhi, India

**Keywords:** Anti-angiogenic, pro-apoptotic, cytotoxicity

## Abstract

**Context:** Abnormal angiogenesis and evasion of apoptosis are hallmarks of cancer. Accordingly, anti-angiogenic and pro-apoptotic therapies are effective strategies for cancer treatment. Medicinal plants, namely, *Eugenia jambolana* Lam. (Myrtaceae), *Musa paradisiaca* L. (Musaceae), and *Coccinia indica* Wight & Arn. (Cucurbitaceae), have not been greatly investigated for their anticancer potential.

**Objective:** We investigated the anti-angiogenic and pro-apoptotic efficacy of ethyl acetate (EA) and *n*-butanol (NB) extracts of *E. jambolana* (seeds), EA extracts of *M. paradisiaca* (roots) and *C. indica* (leaves) with respect to mammary neoplasia.

**Materials and methods:** Effect of extracts (2–200 μg/mL) on cytotoxicity and MCF-7, MDA-MB-231 and endothelial cell (EC) proliferation and *in vitro* angiogenesis were evaluated by MTT, ^3^[H]thymidine uptake and EC tube formation assays, respectively. *In vivo* tumour proliferation, VEGF secretion and angiogenesis were assessed using the Ehrlich ascites tumour (EAT) model followed by rat corneal micro-pocket and chicken chorioallantoic membrane (CAM) assays. Apoptosis induction was assessed by morphological and cell cycle analysis.

**Results:** EA extracts of *E. jambolana* and *M. paradisiaca* exhibited the highest cytotoxicity (IC_50_ 25 and 60 μg/mL), inhibited cell proliferation (up to 81%), and tube formation (83% and 76%). *In vivo* treatment reduced body weight (50%); cell number (16.5- and 14.7-fold), secreted VEGF (∼90%), neoangiogenesis in rat cornea (2.5- and 1.5-fold) and CAM (3- and 1.6-fold) besides EAT cells accumulation in sub-G1 phase (20% and 18.38%), respectively.

**Discussion and conclusion:** Considering the potent anti-angiogenic and pro-apoptotic properties, lead molecules from EA extracts of *E. jambolana* and *M. paradisiaca* can be developed into anticancer drugs.

## Introduction

Angiogenesis involves the formation of new vasculature from the already existing blood vessels and is a characteristic phenomenon in numerous diseases, such as tumour formation, rheumatoid arthritis, diabetic retinopathy and psoriasis to name a few (Yu et al. 2009; Miao et al. 2011). This physiological process is effected by various factors such as vascular endothelial growth factor (VEGF), angiopoietins (Ang), platelet-derived growth factor (PDGF), matrix metalloproteinase (MMP) which expedite cell proliferation, tube formation and migration of endothelial cells (Carmeliet & Jain 2011; Wang et al. 2013). There is a rapid increase in angiogenesis when a tumour transits into the malignant state as a result of a process called the ‘angiogenic switch’ (Guo et al. 2013). VEGF-A is one of the critical factors responsible for augmentation of the tumour vascular bed, which is characterized by abnormal features such as high turnover of neovessels, poor perfusion and increased leakage (Claesson-Welsh & Welsh 2013). VEGF is overexpressed in hypoxic tumour cells (Carbajo-Pescador et al. 2013); endothelial cells (ECs) and tumour-associated macrophages (TAMs) (Guo et al. 2013). The majority of studies have shown that VEGF signalling in tumour cells is autocrine in nature, characteristic of more aggressive cancers, although paracrine signalling also occurs (Goel & Mercurio 2013).

Breast cancer, being the second most common malignancy worldwide, with 1.7 million new cases in 2012 (Ferlay et al. 2015), is known to have a poor prognosis due to the specific pattern of metastasis (Steeg 2006). High levels of VEGF mRNA or VEGF have been reported in invasive ductal breast carcinoma compared to benign or normal tissue (Shivakumar et al. 2009; Ali et al. 2011). Among all the other types of invasive ductal breast carcinomas, the most aggressive, highly metastatic and challenging to treat is the triple-negative breast cancer (TNBC) (Fan et al. 2006), which cannot be treated with targeted therapy such as herceptin or tamoxifen and is bound to have the worst prognosis (Yin et al. 2009). However, VEGF has become a potential therapeutic target in a number of solid malignancies, including TNBC. Clinical trials are now evaluating the potential therapeutic agents that either inhibit VEGF or block VEGFR-2 (Dent 2009; Lu et al. 2016). Thus, targeting tumour growth by angiotherapy is considered as one of the best strategies for anticancer therapy (Bikfalvi & Bicknell 2002), thereby the problems of cytotoxicity and chemo-resistance associated with the classical chemotherapies can be overcome to a certain extent.

Apart from antagonizing VEGF, small molecules such as vatalanib, tivozanib, cediranib, and lenvatinib have been shown to inhibit receptor tyrosine kinase (RTK) signalling (Hojjat-Farsangi 2014). A wide variety of compounds of plant origin has been reported to exhibit anti-angiogenic activity through various molecular pathways. Development of new small molecules is based on the discovery of bioactive secondary plant metabolites and new phytochemical drugs that target tumour angiogenesis and induce apoptosis in cancer cells. Plant polyphenols, catechins, flavonoids, terpenes, tannins, alkaloids and polyacetylenes comprise the natural anti-angiogenic phytochemicals (Lu et al. 2016). Compounds such as taxol, camptothecin and combretastatin have been reported to have potent anti-angiogenic properties (Fan et al. 2006). Further, anti-angiogenic effects through inhibition of VEGF signalling have been reported from dietary functional foods such as genistein from soybean, epigallocatechin gallate from green tea, and resveratrol from red grapes. With respect to cancer therapy, a number of important new commercialized drugs have been synthesized, by structural modification of natural compounds (Gordaliza 2007).

This study was conducted to validate the effectiveness of complementary and alternative medicine by evaluating the anti-angiogenic properties of four plant extracts; ethyl acetate (EA) and *n*-butanol (NB) fractions from seeds of *Eugenia jambolana* Lam. (Myrtaceae), EA fraction from the roots of *Musa paradisiaca* L. (Musaceae) and EA fraction from leaves of *Coccinia indica* Wight & Arn. (Cucurbitaceae). Although our previous studies have reported the antihyperglycemic and antioxidative bioactivities of these plant sources (Chatterjee et al. 2009), various other studies have reported a myriad of biological activity that also includes a potent anticancer activity (Baliga 2011; Pekamwar et al. 2013; Nadumane & Timsina 2014). In order to study the role of these extracts on tumour growth and inhibition of VEGF-induced angiogenesis *in vitro*, two invasive ductal breast carcinoma cell lines, namely, MCF-7, MDA-MB-231 (triple-negative) and human umbilical vein endothelial cells (HUVEC) were used. Ehrlich ascites tumour (EAT), a murine mammary carcinoma model was used for *in vivo* studies. In the current study, we report the effect of plant extracts on tumour burden, ascites volume, tumour cell number, peritoneal angiogenesis, and VEGF secretion in ascites. Besides, the anti-angiogenic property of the plant extracts has also been evaluated in a non-tumour context using corneal micro-pocket and CAM assays. Experiments to evaluate the mode of EAT cell death was studied by nuclear staining and cell cycle analysis. Comprehensively, out of the four extracts, our data suggests that two extracts, namely, EA fraction of *E. jambolana* and EA fraction of *M. paradisiaca* have promising anti-angiogenic and pro-apoptotic activity. Further, the active molecules from these plant extracts are potential candidates for developing alternative and complementary treatments for breast cancer patients.

## Materials and methods

### Plant material collection and extraction

Fresh seeds, leaves and roots of *E. Jambolana*, *M. paradisiaca* and *C. indica* were collected from rural areas of Paschim Midnapur District, West Bengal, India in May–July 2012. Identification of the plants was made and a voucher specimen (HPCH No. 8, 7 and 6) was deposited in the Botany Department, Vidyasagar University, Midnapur, India. The plant materials were separated washed thoroughly first with tap water then with deionized water and dried in an incubator completely at 37 °C. About 900 g of dried seeds were collected from 1 kg fresh seeds, 200 g of dried leaves were collected from 1 kg of fresh leaves and 650 g of dried roots were collected from 1 kg of fresh roots and pulverized separately in an industrial electrical grinder. The pulverized material was macerated in separate 20 L percolators with hydro-methanol solvent (H_2_O: MeOH:: 40:60, v/v) (for each 50 g of plant part used at least 250 mL of solvent) at 35 °C with an intermittent stirring for the first 2 h and left for 36 h at 37 °C. This extraction process was repeated four times using freshly prepared hydro-methanol solvent and the final extracts were collected on the 4th day. The extracts were then filtered first through cotton filter followed by No. 1 Whatman filter paper. The hydro-methanol filtrates were vacuum evaporated using Rotavapor (HAHN-SHIN, HS-2000NS, Hahn-Shin Scientific Co., Korea) at 38 °C and lyophilized on bench top K Lyophilizer and finally stored in amber glass containers refrigerated under vacuum for subsequent fractionation.

These dried hydro-methanol extracted powders were subjected to fractionation with laboratory grade solvents (non-polar to polar), dried under partial vacuum at 38 °C to collect the solvent free residues. Each fraction was stored in amber glass containers at 4 °C for experimental use. The extract preparation and fractionation were performed following the method described earlier (Wagner & Bladt 1996) with slight modification.

### Preparation of the plant extracts

The plant extracts (5 mg); EA and NB fractions from seeds of *E. jambolana*; EA fractions from the roots of *M. paradisiaca* and leaves of *C. indica* were dissolved in 0.1% dimethyl sulfoxide (DMSO). Then the volume was made up to 5 mL with phosphate buffered saline (PBS) and filter sterilized using a syringe filter (Millipore, 22 microns) to give a final concentration of 1 μg/μL. All extracts were stored at 4 °C.

### Animals, cell lines, and chemicals

Female Albino-Wistar rats (4–6 months old) and Swiss albino mice (6–8 weeks old) were obtained from the animal house, department of studies in Zoology, University of Mysore, Mysuru, India. EAT cells are maintained in our laboratory and are routinely used for *in vivo* transplantation.

All experiments were approved by the Institutional animal care and use committee of the University of Mysore, according to the guidelines from the committee for purpose of control and supervision of experiments on animals (approval number 122/GO/ReBi/S/99/CPCSEA), the government of India. HUVECs and endothelial growth medium (EGM) were obtained from Cambrex Biosciences, Walkersville, MD. MCF-7 and MDA-MB-231 cell lines were from National Centre for Cell Sciences, Pune, India. ^3^[H]thymidine was from Baba Atomic Research Center, Mumbai, India. DMEM, L15, FBS and penicillin-streptomycin were from Invitrogen, Carlsbad, CA. Poly-2 hydroxyethyl methacrylate (poly 2-HEMA) from Sigma-Aldrich, St. Louis, MO, Fertilized chicken eggs were from the government poultry farm, Bangalore, India. Matrigel was from Becton Dickinson Labware, Bedford, MA. Recombinant vascular endothelial growth factor (rVEGF_165_) was expressed and purified in-house using the pET-3d-VEGF plasmid. All the other reagents were of the highest analytical grade. 

### MTT assay

MDA-MB-231 cells, growing in exponential phase were seeded into 96-well plates (Nunc MicroWell™) in triplicates at an initial density of 3 × 10^4^ cells per well in 100 μL of complete medium. Following 16 h incubation, filter sterilized plant extracts were added to the culture medium at 2, 5, 10, 20, 40, 80, 100, and 200 μg/mL concentrations with appropriate controls and blanks. Cells were incubated for 24 h. Later, 20 μL MTT (5 mg/mL) reagent (Chemicon International, Inc., Temecula, CA) was added and incubated for 4 h at 37 °C. For solubilization of the resultant formazan product, 100 μL of DMSO was added. The absorbance was measured using a multimode reader at a test wavelength of 570 nm and a reference wavelength of 630 nm. (Varioskan™ Flash Multimode Reader, Thermo Scientific). Data obtained are expressed as the percentage of control mean ± SEM of triplicate values. 

### Cell proliferation assay using ^3^[H]thymidine

^3^[H]thymidine incorporation assay was performed as described previously (Raj et al. 2016). To study the *in vitro* effect of plant extracts on the proliferation of HUVEC, MCF-7 and MDA-MB-231 cells, (3 × 10^4^) were seeded into separate 12 well plates in their respective growth medium and were grown for 48 h. On the third day, cells were serum starved for 24 h in media containing 0.1% serum and treated with plant extracts (20 μg/mL) along with rVEGF (10 ng). The negative control wells were vehicle (0.1% DMSO) treated while the positive control wells were rVEGF (10 ng) treated. ^3^[H]thymidine (1 μCi/mL), was added to each well and incubated for 4 h. The cells were washed with PBS, high molecular weight DNA was precipitated using 10% trichloroacetic acid at 4 °C for 30 min. After two washes with ice-cold PBS, the pellet was solubilized in 0.2 N NaOH and 0.1% SDS and taken into scintillation vials containing 5 mL of scintillation cocktail. The incorporated ^3^[H] radioactivity was measured using a liquid scintillation counter (Perkin Elmer Tri-Carb 2900 TR model, Shelton, CT). All the data expressed as the percent mean ± SEM of triplicate values in comparison to positive control.

### In vitro tube formation assay

Tube formation of HUVEC was performed to assess the effect of plant extracts on *in vitro* angiogenesis as described previously (Ramachandra et al. 2009). A flat bottomed 96 well plate (Nunc) was coated with 50 μL of Matrigel and allowed to solidify at 37 °C for 1 h. HUVECs (5 × 10^3^) were seeded on the Matrigel in 100 μL complete EGM medium containing vehicle (0.1% DMSO) for the negative control, rVEGF (10 ng) for the positive control and the four plant extracts (20 μg/mL) along with rVEGF (10 ng) for tests were incubated for 24 h. After incubation_,_ the enclosed networks of complete tubes formed were quantified by enumerating the network branch points formed in randomly chosen fields by photographing at 200 × magnification under an Olympus inverted microscope (CKX40; Olympus, New York). The average branch points were counted using image J1.49 u software (National Institutes of Health, Bethesda, MD).

### Anti-angiogenic effect of plant extracts treatment in vivo

Ehrlich ascites tumour is derived from a murine mammary carcinoma and is an experimental model for breast cancer. EAT cells (5 × 10^6^) were injected intraperitoneally (i.p.) into mice (5 groups, 5n) and tumour growth was recorded every day from the day of transplantation. To verify whether the plant extracts inhibit tumour growth and angiogenesis mediated by EAT cells *in vivo*, the four different extracts (100 mg/kg body weight/day) was injected into the peritoneum of the respective groups of EAT bearing mice every day from the 6th day of transplantation. The weights of the mice were monitored from the 1st day till the 12th day. On the 12th day, the animals were sacrificed and saline (2 mL) was injected (i.p.), and a small incision was made in the abdominal wall to harvest the tumour cells along with ascites fluid, centrifuged at 3000 *g* for 10 min. The volume of ascites formed in both untreated and treated mice was recorded. The pelleted EAT cells were counted by trypan blue dye exclusion method using a hemocytometer. The animals were dissected and the exposed peritoneum was examined for neovascularisation and photographed.

### VEGF-enzyme linked immunosorbent assay (VEGF-ELISA)

Recombinant human VEGF_165_ was used to set up the standard curve. An indirect VEGF-ELISA was performed using ascites fluid harvested from untreated tumour bearing mice as well as plant extracts treated mice as described previously (Lingaraju et al. 2008). In brief, 100 μL of 1:1000 diluted ascites sample from all the treated or untreated mice were coated onto 96-well microplate using carbonate-bicarbonate coating buffer (pH 9.6) at 4 °C overnight. Wells were washed with PBS and blocked with blocking buffer (5% skimmed milk powder in PBS) for 2 h at 37 °C, followed by incubation with the anti-VEGF_165_ primary antibody (1:1000). After 2 h incubation, wells were washed, 100 μL/well of goat anti-rabbit IgG conjugated to alkaline phosphatase secondary antibody (1:2000) was added. Post 2 h incubation; wells were washed prior to addition of 100 μL of the substrate, p-nitrophenyl phosphate (p-NPP). After 30 min, the reaction was terminated by adding 0.1 N NaOH; the absorbance was read at 405 nm using the Infinite 200 PRO multimode plate reader (Tecan, Männedorf, Switzerland).

### Microvessel density scoring

For the histological studies of peritoneal neovasculature, the peritoneal tissue was excised from all the mice of each group and fixed in 10% formalin solution. Microtome sections (5 μm) were made from paraffin embedded peritoneum and stained with hematoxylin and eosin. Microvessel density was assessed using a bright field microscope in 10 fields of the vascularized areas under high power (40 ×) and the average MVD/HPF was noted and photographed at 40 × magnification.

### Rat corneal micro-pocket assay

The corneal micro-pocket assay was performed as described by Nagaraj et al. (2015) to assess the anti-angiogenic effect of plant extracts *in vivo*. Briefly, poly 2-HEMA pellets were formulated in ethanol 12.5% W/V. Aliquots of 10 μL of this solution was taken on a Teflon sheet and vehicle (0.1% DMSO) for the negative control group, rVEGF (10 ng) for positive control group and different plant extracts (20 μg/mL) along with rVEGF (10 ng) for the test group were placed onto the pellet and dried overnight at 4 °C. Wister rats weighing 300–350 g were anesthetized with an i.p. injection of ketamine (87 mg/kg) and xylazine (13 mg/kg). A corneal micro-pocket was made using a sterile scalpel, with the pocket’s base 1 mm from the limbus. A single pellet was advanced into the lamellar pocket to the limbus using corneal forceps. Postoperatively, gentamicin eye drops were applied onto the operated eye. On the 7th day, the rats were anesthetized again and the corneas were photographed using a stereo binocular microscope with CCD camera attachment, Stereo Discovery V20, Carl Zeiss, Germany. The lengths of blood vessels from the limbus were measured using image J1.49 u software (National Institutes of Health, Bethesda, MD).

### Shell-less CAM

The shell-less CAM assay was performed according to Nataraj and Salimath (2013) to study the effect of the plant extracts on the neovasculature. Fertilized chicken eggs were surface sterilized with 70% alcohol and incubated at 37 °C (fan-assisted humidified incubator). Eggs were gently rolled periodically. On day 4, eggs were cracked open and placed onto the cling film hammocks aseptically in a laminar hood. Egg preparation was covered with a sterile Petri dish and transferred to humidified incubator at 37 °C for 48 h. On day 6, sterile filter discs (2 mm), saturated with the plant extracts (20 μg) along with rVEGF (10 ng), rVEGF (10 ng) alone or vehicle (0.1% DMSO) were placed directly over a blood vessel and placed back in the incubator. After 72 h of incubation, the membrane was examined for inhibition of neovascularization. The CAM was photographed under a stereo binocular microscope at 10 × magnification and the total neovascular blood vessels per unit square millimeter (mm^2^) were quantified using Image J1.49 u software (National Institutes of Health, Bethesda, MD).

### Acridine orange-ethidium bromide nuclear staining

Analysis of morphological changes to assess the mode of cell death in the *in vivo* treated EAT cells was performed using acridine orange and ethidium bromide (AO/EtBr) dual staining. Briefly, EAT cells were harvested from all the groups of mice post-treatment as mentioned earlier and first washed with 0.4% ammonium chloride solution to remove RBCs, followed by two rounds of PBS washes. 1 mg/mL AO/EtBr mixture (10 μL) was added to the cell pellet in PBS and 20 μL of cells were placed on a microscopic slide with a cover slip and immediately analyzed under a fluorescence microscope with the fluorescein filter at 40 × magnification (Zeiss AxioVert). Nuclei were visualized; apoptotic cells in ten random fields were counted and photographed.

### Flow cytometric analysis of cell cycle and apoptosis

EAT cells (3 × 10^6^) were seeded in a 6 well plate in complete DMEM media and treated with plant extracts (20 μg/mL) for 24 h at 37 °C and 5% CO_2_ along with vehicle (0.1% DMSO) treated control. Post-treatment, the cells were washed with PBS and fixed overnight in ice-cold 70% ethanol. After fixation, 1 × 10^6^ cells were washed twice with PBS, and the cells were resuspended in the propidium iodide (PI) staining buffer (25 μg/mL PI; 40 μg/mL RNase and 0.03% Igepal in PBS) and analyzed after 30 min in FACS Calibur flow cytometer (Becton Dickinson, San Jose, CA). Cells were analyzed for DNA content in triplicates and the fraction of every cell cycle phases including the sub G1 phase was determined.

### Determination of total phenol and flavonoids contents in the effective plant extracts

The Folin-Ciocalteu’s assay was performed to measure the total amount of polyphenol content (Andzi-Barhé et al. 2015). *Eugenia jambolana* seed extract (0.25 mL) and *M. paradisiaca* root extract (1 mg/mL) were mixed with 1.25 mL Folin–Ciocalteu reagent (0.2 N diluted in methanol). Methanol was used as a reagent blank. After 5 min incubation at room temperature, 1 mL of sodium carbonate solution (75 g/L) was added. The absorbance was measured at 765 nm after 2 h incubation at room temperature. The total phenol content was expressed as mg of Gallic acid equivalents (GAE) per 100 g of extract.

Quantification of flavonoids was performed using aluminium chloride (Andzi-Barhé et al. 2015). Different extracts (1 mg/mL) were mixed in 1 mL of AlCl_3_ (2%). The absorbance was measured at 415 nm after 10 min of incubation. Results were expressed as mg of Quercetin equivalents (EQ) per 100 g of extract.

### Statistics

All the experiments were performed in triplicate with a minimum of three replicates. The means and standard deviations were calculated and values are expressed as mean ± SEM. The significance of the differences among the treatments was determined using one-way analysis of variance (ANOVA). *p*-Values lower than 0.05 (*p* < 0.05) were considered statistically significant and those lower than 0.01 (*p* < 0.01) were considered extremely significant.

## Results

### Cytotoxicity of extracts

**Figure 1.**
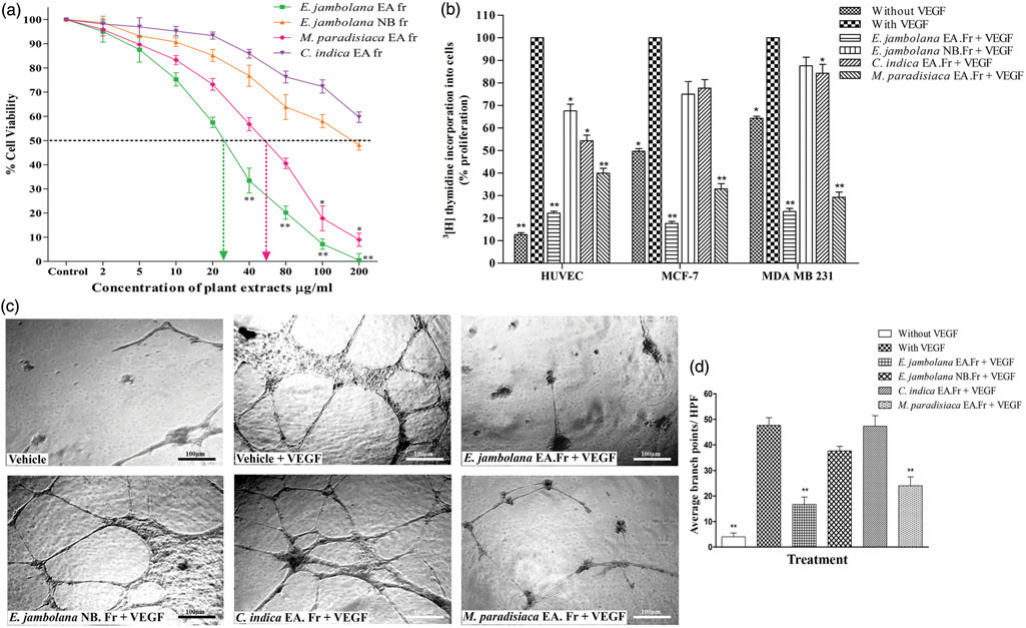
Effects of *E. jambolana, M. paradisiaca,* and *C. indica* extracts on cell proliferation. (a) The metabolic and cytotoxic response of MDA-MB-231 cells assessed by MTT assay. MDA-MB-231 cells (3 × 10^4^) were treated with plant extracts (2, 5, 10, 20, 40, 80, 100, and 200 μg/mL) for 24 h, washed with PBS, incubated for 4 h with MTT and the formazan crystals dissolved in DMSO and read at 570 nm. (b) Cell proliferation assessed by the rate of DNA synthesis using ^3^[H]thymidine. HUVEC, MCF-7 and MDA-MB-231 cells (3 × 10^4^) were cultured in 12-well plates, serum starved and treated with plant extracts (20 μg/mL) along with rVEGF (10 ng) for 24 h. Post incubation for 4 h in presence of ^3^[H]thymidine, the DNA was harvested and rate of cell proliferation was measured in a liquid scintillation counter. (c) HUVEC tube formation assay. HUVECs (5 × 10^3^) were cultured in EGM on Matrigel with plant extracts (20 μg/mL) along with rVEGF (10 ng), in a 96 well plate. After incubation for 24 h at 37 °C, capillary networks were photographed and quantified (Magnification: 200 ×). (d) Quantification of angiogenesis by counting the average number of branch points. Data in all results presented as mean ± SEM of three independent experiments; **p < 0.01 and *p < 0.05.

The effect of different plant extracts on the metabolic activity and consequently the cytotoxicity was assessed by the MTT assay in MDA-MB-231 cells. Increasing concentrations of the four plant extracts were used and the effective concentration was calculated from the dose-response curve along with the IC_50_ values. The percentage cytotoxicity in comparison to the vehicle treated control is shown in Figure 1(a). The EA fraction of *E. jambolana* and the EA fraction of *M. paradisiaca* exhibited significant cytotoxicity with an IC_50_ value of 25 and 60 μg/mL, respectively. On the contrary, NB fraction of *E. jambolana* and the EA fraction of *C. indica* did not exhibit any substantial cytotoxicity.

### Inhibition of cell proliferation

HUVEC, MCF-7 and MDA-MB-231 treated with or without the four plant extracts (20 μg/mL) along with rVEGF (10 ng) for 24 h resulted in considerable decrease in DNA synthesis as measured by the incorporation of ^3^[H]thymidine, which was prominent in cells treated with two of the plant extracts namely the EA fraction of *E. jambolana* and the EA fraction of *M. paradisiaca* as shown in Figure 1(b). Significant inhibition of up to 71.2% and 61.6% in HUVEC, 81% and 71% in MCF-7 and 77% and 75% in case of MDA-MB-231 cells proliferation was observed, respectively.

### Suppression of in vitro angiogenesis

The anti-angiogenic potential of all four plant extracts was evaluated by the *in vitro* tube formation assay, mediated by HUVECs. In the positive control wells with vehicle (0.1% DMSO) and rVEGF (10 ng), HUVECs differentiated into an extensive and enclosed network of tubes. However, the treatment with plant extracts inhibited the tube formation even in presence of rVEGF as shown in Figure 1(c,d). The degree of HUVEC mediated angiogenesis was assessed based on the number of branch points by the image J analysis, which revealed that among all the four extracts the EA fraction of *E. jambolana* and EA fraction of *M. paradisiaca* showed sparse tube networks and acute inhibition of tube formation by 83 and 76%, respectively, compared to rVEGF treated positive control.

### Inhibition of tumour growth and neoangiogenesis in vivo

**Figure 2.**
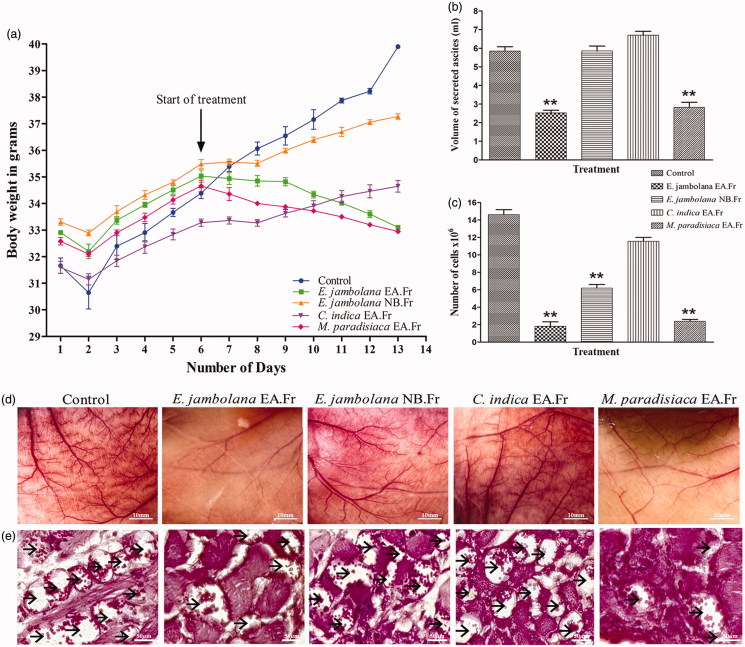
Effects of plant extracts on tumour growth and angiogenesis *in vivo*. (a) Body weights of EAT-bearing; untreated and treated with plant extracts were recorded. 6^th^ day onwards, plant extracts (100 mg/kg body weight) was administered (*i.p*) for six days. The animals were sacrificed on the 12^th^ day. EAT cells were collected along with ascites fluid and centrifuged, (b) secreted ascited volume, (c) EAT cell count assessed by trypan blue dye exclusion method, (d) representative photographs of the peritoneum of the untreated and treated mice and (e) peritoneal MVD marked by arrows in each peritoneum assessed by H and E staining of sections. At least five mice were used in each group and the results obtained are an average of three individual experiments and means of ± S.E.M. *n* = 5 per group.

According to the results in Figure 2(a–c), the vehicle treated EAT bearing control mice showed a steady increase in body weight (8–10 g), the volume of ascites fluid secreted as well as the EAT cell number over a span of 12 days. However, the mice in the treatment groups, specifically the treatment with EA fraction of *E. jambolana* and EA fraction of *M. paradisiaca* exhibited almost 50% reduction in body weight compared to control indicating the effectiveness of the extracts in the prevention of EAT tumour growth. Concurrently a 2.3- and 2.2-fold reduction in secreted ascites fluid along with 16.5- and 14.7-fold reduction in viable cells in comparison to control was observed post-treatment with the above-mentioned extracts, respectively. Although the NB fraction of *E .jambolana* and EA fraction of *C. indica* did show a reduction in body weight, ascites volume, and cell number, it was not on par with that of EA fraction of *E. jambolana* and EA fraction of *M. paradisiaca*.

### Decreased microvessel density (MVD)

An acute reduction in peritoneal angiogenesis was conspicuous in treated groups of mice compared to control EAT bearing mice, shown in Figure 2(d). The smaller capillaries and arterioles were affected by the treatment suggesting inhibition of tumour neoangiogenesis.

Further authentication of the angio-inhibitory effect of the compounds was assessed by histological examination of the peritoneum sections by H and E staining and microscopic examination of the microvasculature. From the photomicrographs (40 × magnification), (Figure 2(e)), it is evident that the MVD is drastically reduced in the peritoneum of mice treated with the EA fraction of *E. jambolana* and EA fraction of *M. paradisiaca* compared to control or other treatments.

### Angio-inhibitory effect of plant extracts

**Figure 3.**
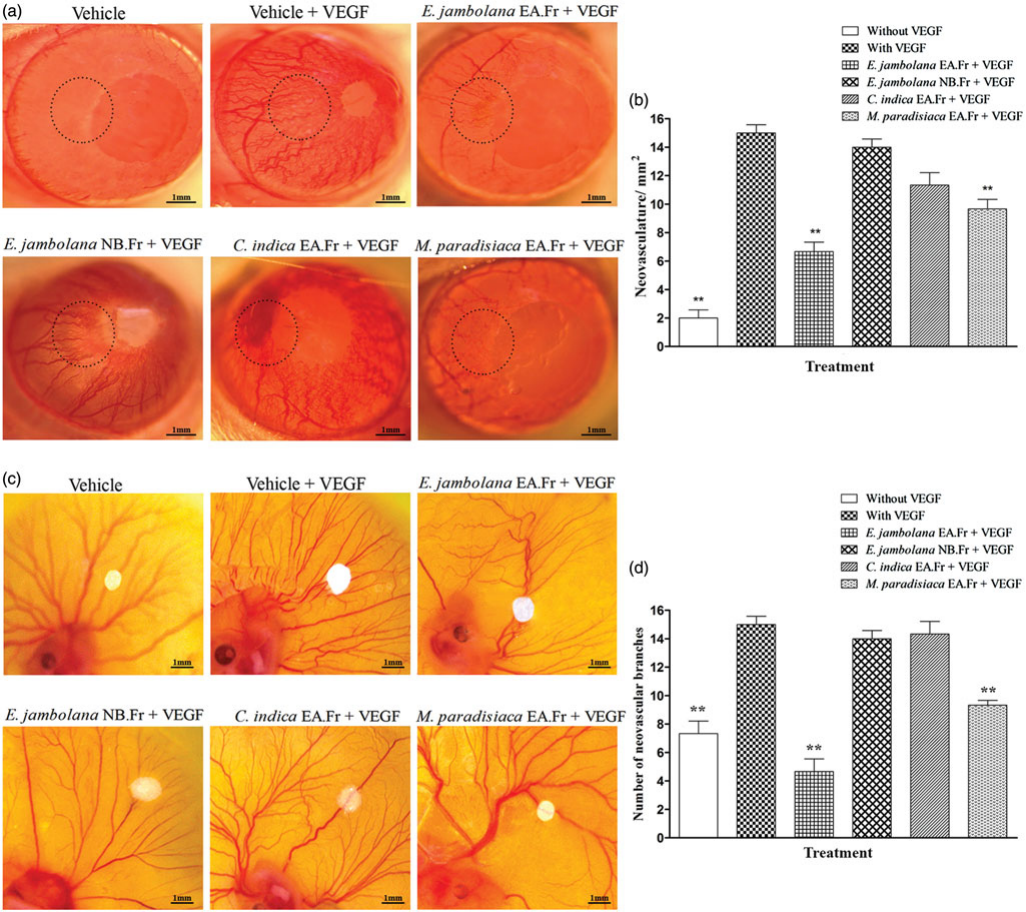
Effects of plant extracts on neovasculature in the rat cornea and chick embryo CAM assays. (a) Representative photographs of VEGF-induced rat corneal neovascularization, plant extracts 20 μg/hydron polymer pellet were surgically implanted into the micro-pocket in the cornea of one eye. On day 7, the extent of neovascularization or inhibition was visualized and photographed under a dissection microscope. (b) Quantitative comparison of the number of neovascular vessels per mm^2^ was estimated. (c) Representative photographs of VEGF-induced neovascularization in shell-less CAM of chicken embryos. Plant extracts, 20 μg/filter disc was placed on the CAM of 6-day old chicken embryos. After 72 h of incubation, the area surrounding the filter disc was inspected for changes in neovascularization. (d) Quantitative comparison of the number of neovascular branches surrounding the plant extract containing filter paper discs. The data shown represent the results of experiments that were performed using a maximum of 5 eggs in each group. All quantitative data are presented as mean ± SEM of five independent counts; **p < 0.01.

The rat corneal micro-pocket assay and the shell-less chorioallantoic membrane assay are generally used in the *in vivo* assessment and validation of the angio-inhibitory potential of anti-angiogenic compounds in a non-tumour context. Results (Figure 3(a–d)) clearly indicate that the EA fraction of *E. jambolana* and EA fraction of *M. paradisiaca* are efficient inhibitors of neovasculature even in presence of rVEGF (10 ng) *in vivo*, both in rat cornea and the CAM in comparison to rVEGF (10 ng) treated positive control which showed extensive angiogenesis.

In the rat cornea, the effect of EA fraction of *E. jambolana* and EA fraction of *M. paradisiaca* on the reduction of neovasculature/mm^2^ was 2.5- and 1.5-fold, respectively. Similarly, in the CAM assay, the reduction in the number of vascular branches was 3- and 1.6-fold, respectively.

### Inhibition of VEGF production in EAT cells

**Figure 4.**
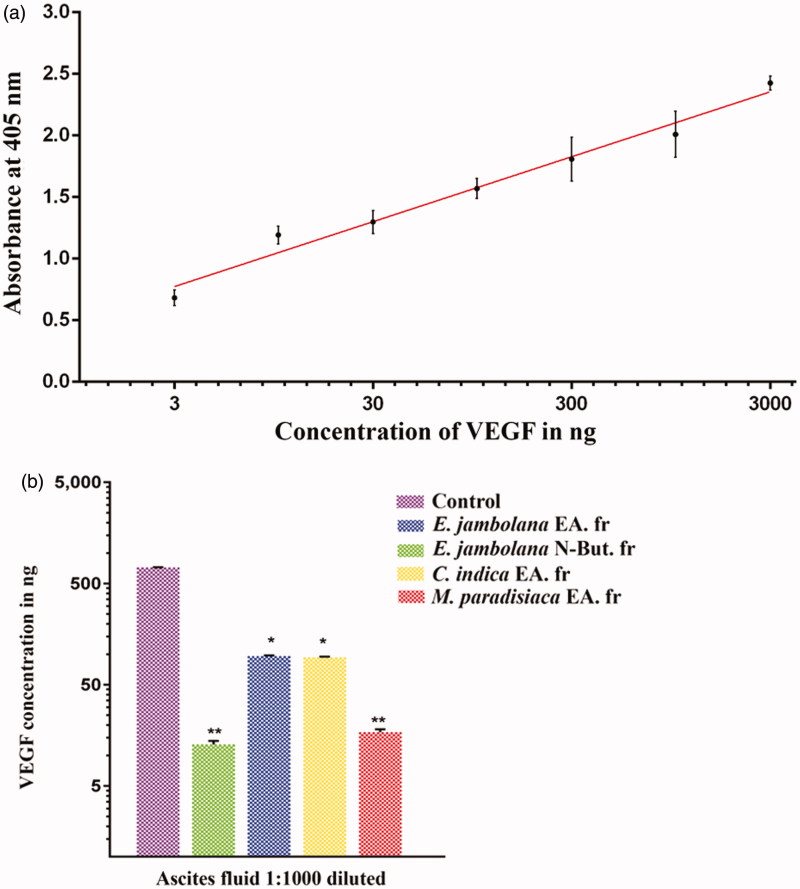
Effects of plant extracts on the secretion of VEGF in ascites fluid of tumour bearing mouse. Indirect ELISA was carried out using the ascites fluid harvested from the control (tumour bearing) and plant extracts treated (100 mg/kg body weight) mice to quantify the VEGF in ascites fluid using anti-VEGF_165_ antibodies. (a) ELISA standard curve for VEGF. (b) The histogram showing a comparison of VEGF levels in the ascites of untreated control and treated groups. Data are representative of three independent experiments and values are expressed in mean ± SEM, **p < 0.01 and *p < 0.05.

The VEGF standard curve was observed to have good linearity as shown in Figure 4(a). The ELISA results showed elevated levels of VEGF in the ascites fluid taken from the tumour-bearing control mice (721 ng). However, a significant decrease (∼90%) in VEGF levels was observed in the groups treated with EA fraction of *E. jambolana* (13 ng) and EA fraction of *M. paradisiaca* (17 ng). But groups treated with NB fraction of *E. jambolana* and EA fraction of *C. indica* showed a comparatively lower decrease in VEGF levels of 96 and 93 ng, respectively, (Figure 4(b)), suggesting the inhibition of VEGF secretion *in vivo*.

### Effects of plant extracts treatment in vivo on EAT cell morphology

**Figure 5.**
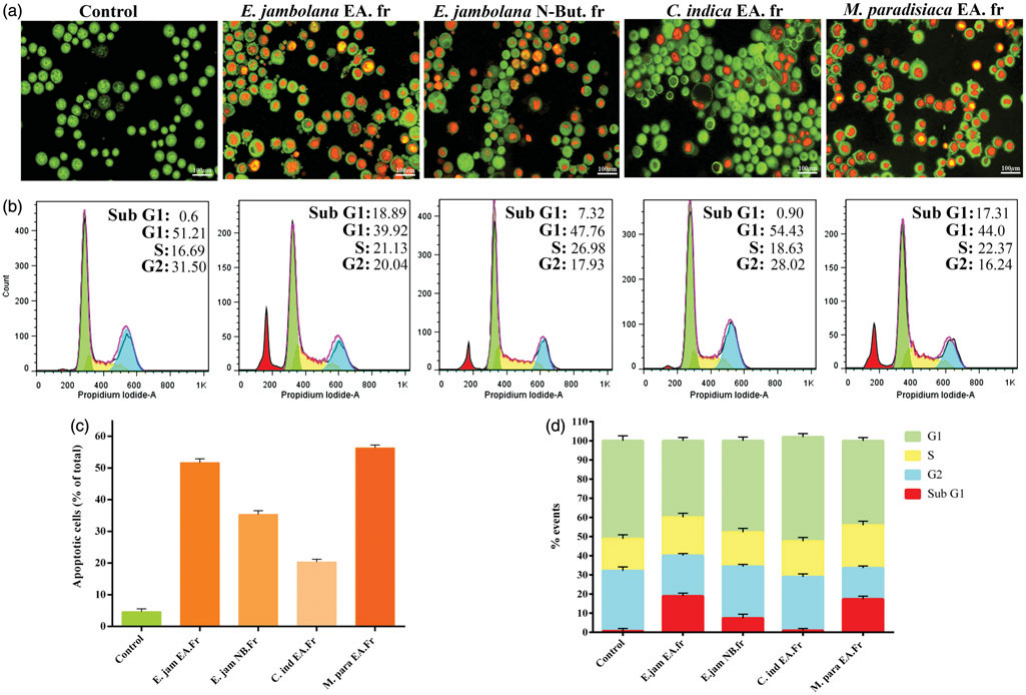
Apoptosis induction and cell cycle analysis of plant extract treated EAT cells. (a) *In vivo* treated EAT cells with or without plant extracts were stained with acridine orange and ethidium bromide and verified for apoptotic characteristics such as plasma membrane blebbing, chromatin condensation and apoptotic body formation under a fluorescence microscope. (b) Effect of plant extracts on cell cycle progression. EAT cells, *in vitro*, were treated with or without different plant extracts (20 μg/mL). After 24 h of treatment, distribution of cell cycle phases and apoptotic cell population was quantitated based on flow cytometric analysis. (c) A quantitative comparison of the number of apoptotic cells in 10 random fields. (d) Bar diagram showing the percentage of cells present in different phases of cell cycle.

EAT cells, post-harvest from treated and control groups were dual stained with acridine orange and ethidium bromide and examined under a fluorescent microscope. No significant morphological changes were observed in the vehicle-treated control cells, most of them appeared green with intact nuclei. However, the cells from the treated groups showed the early and late stages of apoptosis, manifested by the shrunken and crescent-shaped orange nuclei, membrane blebbing and apoptotic bodies containing fragmented nuclei. The percentage of apoptotic cells was determined by counting the number of apoptotic cells under the microscope in ten random fields in comparison to the vehicle treated control. Results, (Figure 5(a,c)) clearly show increased apoptosis induction by 51% and 56% by the EA fraction of *E. jambolana* and EA fraction of *M. paradisiaca* treatments, respectively.

### Plant extracts induce apoptosis without cell cycle arrest

The inhibitory effect of plant extracts on EAT cell proliferation was ascertained by cell cycle analysis by flow cytometry. EAT cells; post-treatment with plant extracts for 24 h, the cell cycle distribution was analyzed by flow cytometry following staining with propidium iodide (PI). A significant accumulation of cells was observed in sub-G1 phase in cells treated with EA fraction of *E. jambolana* (20%) and EA fraction of *M. paradisiaca* (18.38%), characteristic of apoptosis, whereas treatments with NB fraction of *E. jambolana* and EA fraction of *C. indica* showed 7.53% and 0.94%, respectively as shown in Figure 5(b,d). However, no cell cycle arrest was observed upon treatment.

### Total phenolic and flavonoids content

The results (Table 1) revealed that the bioactive EA fraction of seed extract of *E. jambolana* had a comparatively lower content of phenolic compounds (18.54 mg GAE/100 g of extract) in comparison to the flavonoids content (122.32 mg EQ/g of extract). Whereas the EA fraction of root extract of *M. paradisiaca* showed high phenolic compounds content (118 mg GAE/100 g of extract) in comparison to the flavonoids (78 mg EQ/g of extract).

**Table 1.** The phytochemical analysis (total phenol and flavonoids content) of the two plant extracts, namely, EA fraction of *Eugenia jambolana* and EA fraction of *Musa paradisiaca* were performed and the values obtained are as shown.

Plant extract	Total phenolic content (mg GAE/g dried extract	Total flavonoids (μg EQ/g dried extract)
Ethyl acetate fraction of seed of *E. jambolana*	18.54	122.32
Ethyl acetate fraction of root of *M. paradisiaca*	118	78

GAE: Gallic acid equivalents; EQ: Quercertin equivalents; *E. jambolana*: *Eugenia jambolana; M. paradisiaca*: *Musa paradisiaca*.

## Discussion

Sustained angiogenesis, being a hallmark of cancer, has a fundamental role in tumour growth, invasion and metastasis. In pathological conditions like chronic inflammation, diabetic retinopathy, rheumatoid arthritis or atherosclerosis, angiogenesis is typically upregulated (Quesada et al. 2006). Thus, the prime importance and the understanding of new blood vessels formation have led to novel therapies designed to interrupt this process (Miller et al. 2001). As part of our search for a natural product based anti-angiogenic agents, we studied the effects of the four plant extracts; EA and NB fractions from seeds of *Eugenia jambolana*, EA fraction from the roots of *Musa paradisiaca* and EA fraction from leaves of *Coccinia indica*.

Various bioactive chemical compounds of plant origin may influence the angiogenic potential of various cell types and hence upsets the formation of blood vessels (Loboda et al. 2005; Neal et al. 2006). Several alkaloids such as sanguinarine, isolated from the root of *Sanguinaria canadensis* L. (Papaveraceae) and vinca alkaloids obtained from *Catharanthus roseus* (L.) G. Don (Apocynaceae) have been exploited to target angiogenesis (Eun & Koh 2004; Ribatti et al. 2005). Halofuginone, an alkaloid isolated from *Dichroa febrifuga* Lour. (Hydrangeaceae), is a potent inhibitor of crucial steps in angiogenesis cascade (Elkin et al. 2000). The primary strategy for the screening of novel anti-angiogenic compounds is to look for EC growth inhibitors (Quesada et al. 2006). Also, the invasive ability of the EC requires extracellular matrix degradation and involves the activation of many EC signalling pathways which can also be studied. Pharmacologically important bioactive organic compounds such as phenols, flavonoids, tannins, saponins, and terpenes have better solubility in solvents such as ethyl acetate and *n*-butanol. Hence in this study, we screened the plant extracts obtained from the EA and NB fractions. Furthermore, we chose to study the anti-angiogenic property of these extracts based on our previous work on antioxidant and antidiabetic properties of these extracts (Mallick et al. 2006, 2009; Ghosh 2015). The preliminary toxicity profile of all the four plant extracts exhibited cytotoxicity in a dose-dependent manner as assessed by MTT assay. However, EA fraction of *E. jambolana* and EA fraction of *M. paradisiaca* showed high cytotoxicity with lower IC_50_ values compared to NB fraction of *E. jambolana* or EA fraction of *C. indica*. Subsequently, our studies with a subtoxic concentration of all the extracts on the cell proliferation assay using a normal cell line (HUVEC) and two breast cancer cell lines (MCF-7 and MDA-MB-231) revealed a significant reduction in rate of cell proliferation even in the presence of a potent cytokine-VEGF, which may suggest the interference of VEGF signalling by the bioactive constituents in the plant extracts. Numerous plant compounds possess anti-angiogenic property by disrupting angiogenesis signalling cascade (Lee et al. 2015), like the capsaicin, curcumin or quercetin-mediated inhibition of VEGF-induced angiogenesis (Gururaj et al. 2002; Min et al. 2004; Pratheeshkumar et al. 2012).

The inhibition of tumour angiogenesis, growth and metastasis by means of phytochemicals is reportedly due to the regulation of signalling pathways of VEGFR (Fukuda et al. 2006). Thus, to further inspect whether this curtailment in the rate of cell proliferation was directly due to cytotoxicity or an inherent anti-angiogenic property, the *in vitro* endothelial tube formation assay was performed using HUVECs in presence of VEGF. Our results showed that both the EA fraction of *E. jambolana* and EA fraction of *M. paradisiaca* were indeed anti-angiogenic and effectively blocked VEGF-induced angiogenesis *in vitro*.

To further validate these results, the next step was to evaluate the antitumor, anti-proliferative and anti-angiogenic properties of these extracts *in vivo*. Hence, we adopted the murine mammary carcinoma, EAT model for the study. The *in vivo* data showed a drastic reduction in tumour burden, EAT cell number, ascites formation and also secreted VEGF levels which bear significant importance in terms of a clinical correlation with inhibited ascites formation in human tumours. The concomitant reduction in the peritoneal neovasculature and corresponding decrease in the MVD was observed in the peritoneum of the treated mice.

The substantiation of the anti-angiogenic property of the two plant extracts further led us to perform the classical *in vivo* and *ex vivo* angiogenic assays, namely, the rat corneal micro-pocket assay and shell-less CAM assay (Wang et al. 2004), respectively. The results of these two experiments were consistent with the earlier results where the number of new blood vessels was significantly decreased upon treatment with EA fraction of *E. jambolana* or EA fraction of *M. paradisiaca*, in comparison to VEGF treatment that showed increased neoangiogenesis.

Furthermore, apart from the anti-angiogenic activity of the plant extracts, the EAT cells harvested after *in vivo* treatment interestingly showed induction of cell death by apoptosis, which was rather pronounced in the EA fraction of *E. jambolana* and EA fraction of *M. paradisiaca*, confirmed by the AO/EtBr dual stained morphological study and also by the cell cycle analysis. These results suggest that the extracts may also contain potent pro-apoptotic compounds.

The plant extracts showing significant bioactivity could be attributed to the high flavonoid content in *E. jambolana* seeds, since flavonoids have been reported to reduce the risk of development of breast cancer and prostate cancer by induction of apoptosis, reducing oxidative stress (Afsar et al. 2016) and also inhibit VEGF-induced cell proliferation and migration in HUVECs, as well as angiogenesis (Wu et al. 2012). Also, the high phenolic content in the *M. paradisiaca* roots may be contributing to the anti-angiogenic, anti-proliferative and pro-apoptotic properties since phenols have been known to play a critical role in inhibition of VEGF, PDGF receptor phosphorylation, MMP inhibition, cell migration, inhibition of ROS (Sun et al. 2015) and also induction of apoptosis in cancer cells (Roy et al. 2002).

## Conclusions

Taken together, representing the sequential events in the angiogenic process, the two plant extracts, namely, EA fractions of *E. jambolana* seeds and *M. paradisiaca* roots, showed strong anti-proliferative and anti-angiogenic effects in breast cancer cell lines through the inhibition of VEGF-induced cell proliferation, HUVEC tube formation *in vitro* as well as suppression of VEGF-induced rat corneal neovascularization *in vivo* and also CAM neovascularisation *ex vivo*. In addition, in the *in vivo* EAT mouse tumour model, there was a profound reduction in EAT tumour growth, ascites volume, ascites VEGF levels, tumour cell number and also peritoneal angiogenesis. The anti-proliferative effect was evident in the EAT cells, which clearly showed cell death by induction of apoptosis. Thus, in conclusion, all the results advocate an authenticated evidence that the two plant extracts have a potent therapeutic potential in the treatment of cancer or angiogenesis-related disorders. The findings of this study contribute to the pharmacological knowledge and the therapeutic efficacy of *E. jambolana* and *M. paradisiaca*, and can initiate the development of new anti-angiogenic drugs. However, further studies to identify the individual bioactive compounds and their translational research is essential to elucidate their explicit mechanisms.
